# A multi-stakeholder multicriteria decision analysis for the reimbursement of orphan drugs (FinMHU-MCDA study)

**DOI:** 10.1186/s13023-021-01809-1

**Published:** 2021-04-26

**Authors:** Fernando de Andrés-Nogales, Encarnación Cruz, Miguel Ángel Calleja, Olga Delgado, Maria Queralt Gorgas, Jaime Espín, Jorge Mestre-Ferrándiz, Francesc Palau, Alba Ancochea, Rosabel Arce, Raquel Domínguez-Hernández, Miguel Ángel Casado, the FinMHU-MCDA Group, Pedro Gómez Pajuelo, Maria Queralt Gorgas Torner, Antonio López Andrés, Mónica López Rodríguez, Adela Marín Ballvé, María Isabel Martín Herranz, Alberto Morell Baladrón, Fernando Ignacio Sánchez Martínez, Alba Ancochea, Fernando Antoñanzas, Santiago Bonanad, Encarnación Cruz, Teresa Caballero, Juan Manuel Cabasés, Miguel Ángel Calleja, Jordi Cruz, Olga Delgado, Jaime Espín, Manuel García-Goñi, Ricardo Gil, Pedro Gómez Pajuelo, Maria Queralt Gorgas Torner, Antonio López Andrés, Mónica López Rodríguez, Adela Marín Ballvé, María Isabel Martín Herranz, Jorge Mestre-Ferrándiz, Alberto Morell Baladrón, Carlos Mur, Francesc Palau, Matilde P. Machado, Fernando Ignacio Sánchez Martínez, Alba R. Santos, Mónica Suárez, José Luis Trillo

**Affiliations:** 1 Pharmacoeconomics & Outcomes Research Iberia (PORIB), Calle Paseo Joaquín Rodrigo, 4I. 28224 Pozuelo de Alarcón, Madrid, Spain; 2 Asociación Española de Medicamentos Biosimilares, Madrid, Spain; 3 Servicio de Farmacia, Hospital Universitario Virgen Macarena, Sevilla, Spain; 4 Servicio de Farmacia, Hospital Universitario Son Espases, Palma de Mallorca, Spain; 5 Servicio de Farmacia, Hospital Universitari Vall D’Hebron, Barcelona, Spain; 6 Escuela Andaluza de Salud Pública, Granada, Spain; 7 Instituto de Investigación Biosanitaria (IBS), Granada, Spain; 8 CIBER de Epidemiología Y Salud Pública (CIBERESP), Madrid, Spain; 9 Consultor Económico Independiente, Madrid, Spain; 10 Madrid, Spain; 11 Servicio de Medicina Genética y CIBERER, Hospital Universitari Sant Joan de Déu, Hospital Clínic y Universitat de Barcelona, Barcelona, Spain; 12 Federación Española de Enfermedades Raras (FEDER), Madrid, Spain; 13 Asociación Española de Laboratorios de Medicamentos Huérfanos y Ultrahuérfanos (AELMHU), Barcelona, Spain; 14 Universidad de La Rioja, Logroño, Spain; 15 Hospital Universitari i Politècnic La Fe, Valencia, Spain; 16 Hospital Universitario La Paz, IdiPaz, CIBERER U754, Madrid, Spain; 17 Universidad Pública de Navarra, Pamplona, Spain; 18 Asociación de Mucopolisacaridosis España (MPS España), Barcelona, Spain; 19 Hospital Universitario Son Espases, Sociedad Española de Farmacia Hospitalaria, Palma de Mallorca, Spain; 20 Escuela Andaluza de Salud Pública, Centro de Investigación Biosanitaria (IBS) y CIBERESP, Granada, Spain; 21 Universidad Complutense de Madrid, Madrid, Spain; 22 Hospital Universitari i Politècnic La Fe (Medicina Interna), Valencia, Spain; 23 Economista de la Salud, Madrid, Spain; 24 Hospital Universitari Vall d’Hebron, Barcelona, Spain; 25 Subdirección de Farmacia, Servicio Navarro de Salud-Osasunbidea, Pamplona, Spain; 26 Hospital Universitario Ramón y Cajal, Universidad de Alcalá, IRYCIS, Madrid, Spain; 27 Hospital Clínico Universitario Lozano Blesa, Zaragoza, Spain; 28 Instituto de Investigación Biomédica de A Coruña (INIBIC), Complexo Hospitalario Universitario de A Coruña (CHUAC), Universidade da Coruña (UDC), A Coruña, Spain; 29 Hospital Universitario de La Princesa, Madrid, Spain; 30 Consejería de Sanidad, Madrid, Spain; 31 Hospital Universitari Sant Joan de Déu y CIBERER, Barcelona, Spain; 32 Universidad de Murcia, Murcia, Spain; 33 Asociación NUPA, Madrid, Spain; 34 Federación Española de Enfermedades Neuromusculares, Federación ASEM, Barcelona, Spain; 35 Departamento de Salud Clínico Malvarrosa, Valencia, Spain

**Keywords:** Multicriteria decision analysis, Orphan drugs, Rare diseases, Reimbursement, Spain

## Abstract

**Background:**

Patient access to orphan medicinal products (OMPs) is limited and varies between countries, reimbursement decisions on OMPs are complex, and there is a need for more transparent processes to know which criteria should be considered to inform these decisions. This study aimed to determine the most relevant criteria for the reimbursement of OMPs in Spain, from a multi-stakeholder perspective, and using multicriteria decision analysis (MCDA).

**Methods:**

An MCDA was developed in 3 phases and included 28 stakeholders closely related to the field of rare diseases (6 physicians, 5 hospital pharmacists, 7 health economists, 4 patient representatives and 6 members from national and regional health authorities). Initially [phase A], a bibliographic review was conducted to identify the potential reimbursement criteria. Then, a reduced advisory board (8 members) proposed, selected, and defined the final list of criteria that could be relevant for reimbursement. A discrete choice experiment (DCE) [phase B] was developed to determine the relevance and relative importance weight of such criteria according to the stakeholders’ preferences by choosing between pairs of hypothetical financing scenarios. A multinomial logit model was fitted to analyze the DCE responses. Finally [phase C], the advisory board review the results using a deliberative process.

**Results:**

Thirteen criteria were selected, related to 4 dimensions: patient population, disease, treatment, and economic evaluation. Nine criteria were deemed relevant for decision-making and associated with a higher relative importance: Health-related quality of life (HRQL) (23.53%), treatment efficacy (14.64%), availability of treatment alternatives (13.51%), disease severity (12.62%), avoided costs (11.21%), age of target population (7.75%), safety (seriousness of adverse events) (4.72%), quality of evidence (3.82%) and size of target population (3.12%). The remaining criteria had a < 3% relative importance: economic burden of disease (2.50%), cost of treatment (1.73%), cost-effectiveness (0.83%) and safety (frequency of adverse events) (0.03%).

**Conclusion:**

The reimbursement of OMPs in Spain should be determined by its effect on patient’s HRQL, the extent of its therapeutic benefit from efficacy and the availability of other therapeutic options. Furthermore, the severity of the rare disease should also influence the decision along with the potential of the treatment to avoid associated costs.

## Background

Rare diseases pose a threat to the health of individuals. They are diseases of low prevalence and high complexity that can lead to death or chronic disability [1] and for which there frequently are no therapeutic options [2]. These generally serious, chronic, and progressive diseases have a high impact on patients, their families, and even society, and are characterized by pain, disability, significant organ damage, and high mortality rates [3].

In Europe, rare diseases are defined as those pathologies that affect less than 5 people per 10,000 inhabitants [4], most of them being extremely rare or ultrarare, affecting less than 1 person per 50,000 inhabitants [2]. Despite their low frequency, since there are many different rare diseases, they affect millions of people. It is estimated that there are more than 6,000 rare diseases [5], affecting between 6 and 8% of the population at some point in their lives [1]. Because of their low prevalence, their specificity, and the high number of people they affect altogether, these pathologies require a comprehensive approach and priority of action to prevent significant morbidity or premature mortality and to improve the quality of life and socioeconomic potential of the people [1].

Orphan medicinal products (OMPs), which are intended to diagnose, prevent, or treat rare diseases, have a shared community procedure for being designated as such in the European Union, and this community approach provides opportunities for research, development, and marketing [4, 6]. Despite regulations at the European level for centralized approval, patient access to medicines depends on the pricing and reimbursement policies established individually in each member state; and these policies vary widely, given the difficulties of evaluating the available evidence during their pricing and reimbursement process [7, 8]. Financing decisions for OMPs are complex and are conditioned by several conflicting factors, including the promotion of equitable and timely access for patients, cost containment strategies to maintain public services, and the reward for innovation [9, 10].

In Spain, once marketing is authorized by the European Commission and after obtaining a national code, the maximum industrial price and the funding conditions of the medicine are established [11]. Only about half of the OMPs authorized by the European Medicines Agency (EMA) [8, 12] are marketed and reimbursed by the Spanish national health care system, fewer than in other European countries such as Germany, France, and Italy, although similar funding decisions were taken in Spain and Italy compared to between other countries [13]. In Spain, as in other European countries, a conditional approval of the drug by the EMA delays and reduces the likelihood of financing by 80%, which is relevant given that a quarter of the cancer OMPs are conditionally approved and, a third of the OMPs for metabolic diseases are approved under exceptional circumstances [13]. The average time between the European Commission’s marketing authorisation decision and the establishment of the price and reimbursement condition of an OMP in Spain is estimated at 23 months [10, 12], and the period between the authorization at the national level by the Spanish Medicines Agency (AEMPS) (national code assignment) and financing is 13.7 months [10]. After the national pricing and reimbursement process, several autonomous agencies and regional and hospital committees re-evaluate the drug and its clinical conditions of use, which results in the establishment of different access criteria. These differences in the availability of medications have caused many people affected by rare diseases to experience serious difficulties in accessing the treatment they need [3].

In most developed countries, including Spain, OMPs are financed under the same criteria as other medicines [14–16]. Health-technology assessment processes, in addition to evaluating a drug’s efficacy, safety, and quality, usually consider cost-effectiveness in decisions about pricing and reimbursement, but when evaluating treatments for rare diseases, limitations arising from high prices and high uncertainty regarding efficacy make it difficult to reach the established cost-effectiveness thresholds [7, 17]. Therefore, many consider cost-effectiveness analysis insufficient as a basis for these decisions [18]. However, many of the OMPs, despite not being cost-effective, end up being funded, which implies that in practice, criteria besides efficiency are taken into account [16, 18]. Although the criteria that are used in the decision-making process for the reimbursement of drugs in Spain are included in the current legislation [19], their practical implementation is unknown, and the pricing and reimbursement system needs to be more transparent, in line with the recently published recommendations [20].

To establish the key factors in the reimbursement process, it is necessary to develop specific tools that reflect the priorities and perspectives of all the agents involved in decision-making regarding access to, and financing of medicinal products, and especially OMPs. Multicriteria decision analysis (MCDA) is a set of methods and tools that support complex decision-making, overcoming the limitations inherent to treatments with OMPs and improving the technical quality, ethics, and transparency of decisions on prioritization, inclusion in the therapeutic arsenal, and financing of OMPs. These methods provide the analytical capacity and methodological infrastructure necessary to explicitly add all the elements, interests, criteria, values, and concerns on which decision-making is based. In addition, in an MCDA study, the inclusion of participatory processes is critical for incorporating social value into the different dimensions to be considered and for building decision rules [17], as it is important to involve different stakeholders in the process. Beyond establishing whether policymakers consider a particular criterion relevant when making decisions, MCDA studies can determine how much weight or importance they give to one criterion over another [18].

It is difficult to establish a single overall proposal for the implementation of MCDA since each country or region has different values, capacities, resources, and constraints [18]. Therefore, it is necessary to develop specific tools that reflect the priorities and perspectives of the stakeholders involved when considering the factors relevant to OMP access and financing at the national level.

The objective of this study was twofold: first, to review, discuss, and reach a consensus on the most relevant criteria for decision-making about pricing and financing OMPs in Spain; and second, to prioritize them according to their relative importance based on the preferences stated by different stakeholders, following the MCDA methodology.

## Methods

The FinMHU-MCDA study consisted of an MCDA based on the international recommendations of the International Society for Pharmacoeconomics and Outcomes Research (ISPOR), which establishes the appropriate steps and techniques for such analyses [21, 22].

**Fig. 1:**
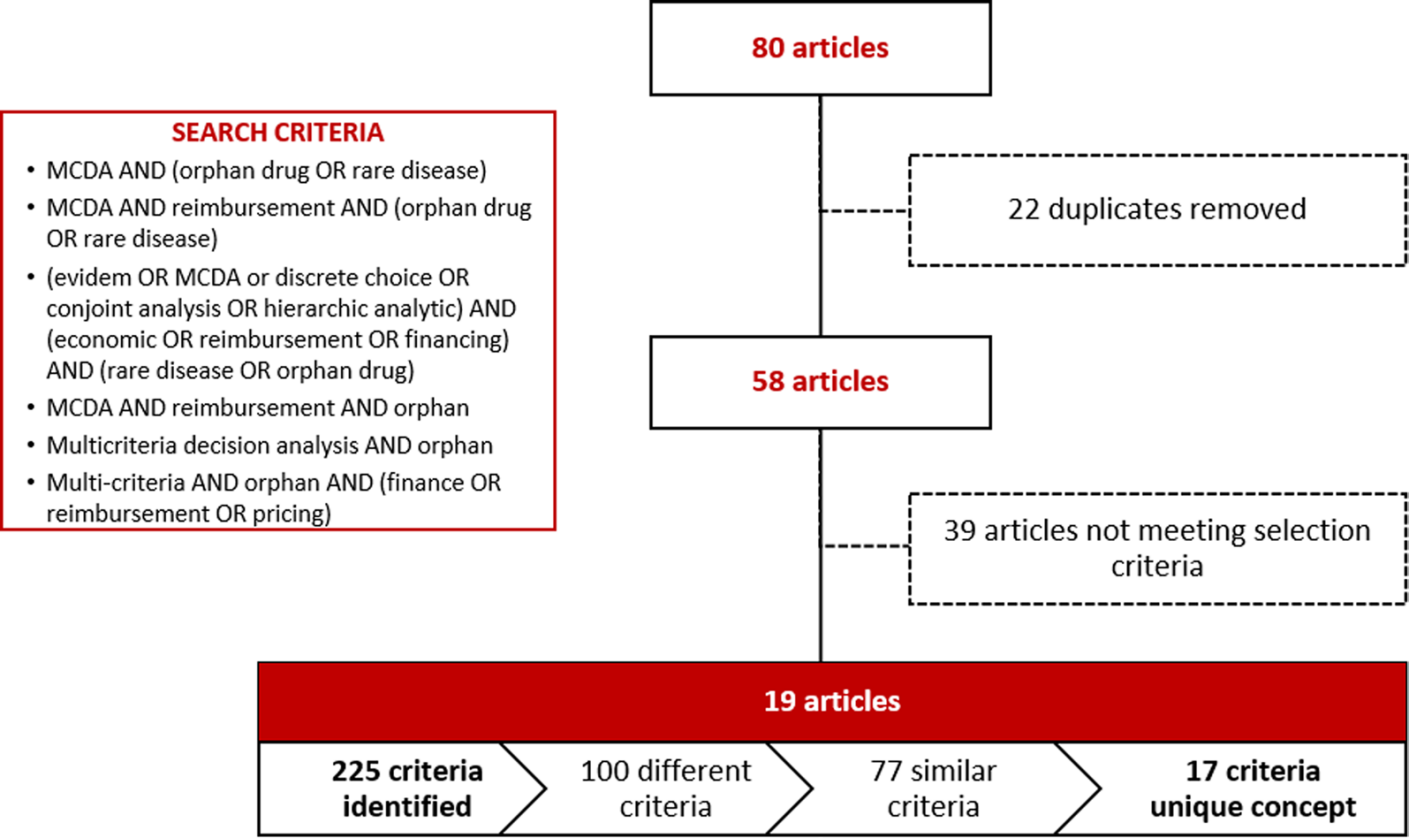
Results of the literature search for the identification of reimbursement criteria

The work of this study was structured into three different phases. In the initial phase (phase A), we chose and defined the criteria to be considered in reimbursement decisions for OMPs. In phase B, the relevant criteria for financing were established based on their relevance for decision-making and then prioritized by weighting them according to their relative importance. Finally, in phase C, the results were discussed through a deliberative process (Fig. 1).

In this project, there were a total of 28 participants included out of 89 contacts who were invited to participate, belonging to sectors with decision-making capacity and holding opinion-leading positions on orphan drugs and rare diseases, including clinical management, health management, and health technology assessment. These participants were classified into five groups according to the type of stakeholder: Health Authorities (six), with national or regional responsibilities and experience; Hospital Pharmacy (five), with experience in the field of OMPs; Health Economics, including university academics and professionals in the sector (seven); Clinicians, including physicians with experience and knowledge in the management of patients with rare diseases (six); and representatives of Patient Associations (four). Prior the selection of the rest of the participants, a panel of eight experts was chosen to participate in the face-to-face phases (A and C) of the study. This panel included three hospital pharmacists, two health economists, a physician specializing in rare diseases, a representative of health authorities, and a representative of the Spanish Alliance on Rare Diseases (*Federación Española de Enfermedades Raras*, FEDER).

### Phase A: selection and definition of criteria

The objective of this phase was to identify, select, and define the criteria relevant to the financing of OMPs that would serve as the basis for the following phases of the study. This phase included an online questionnaire and a face-to-face meeting of the panel of experts.

**Fig. 2:**
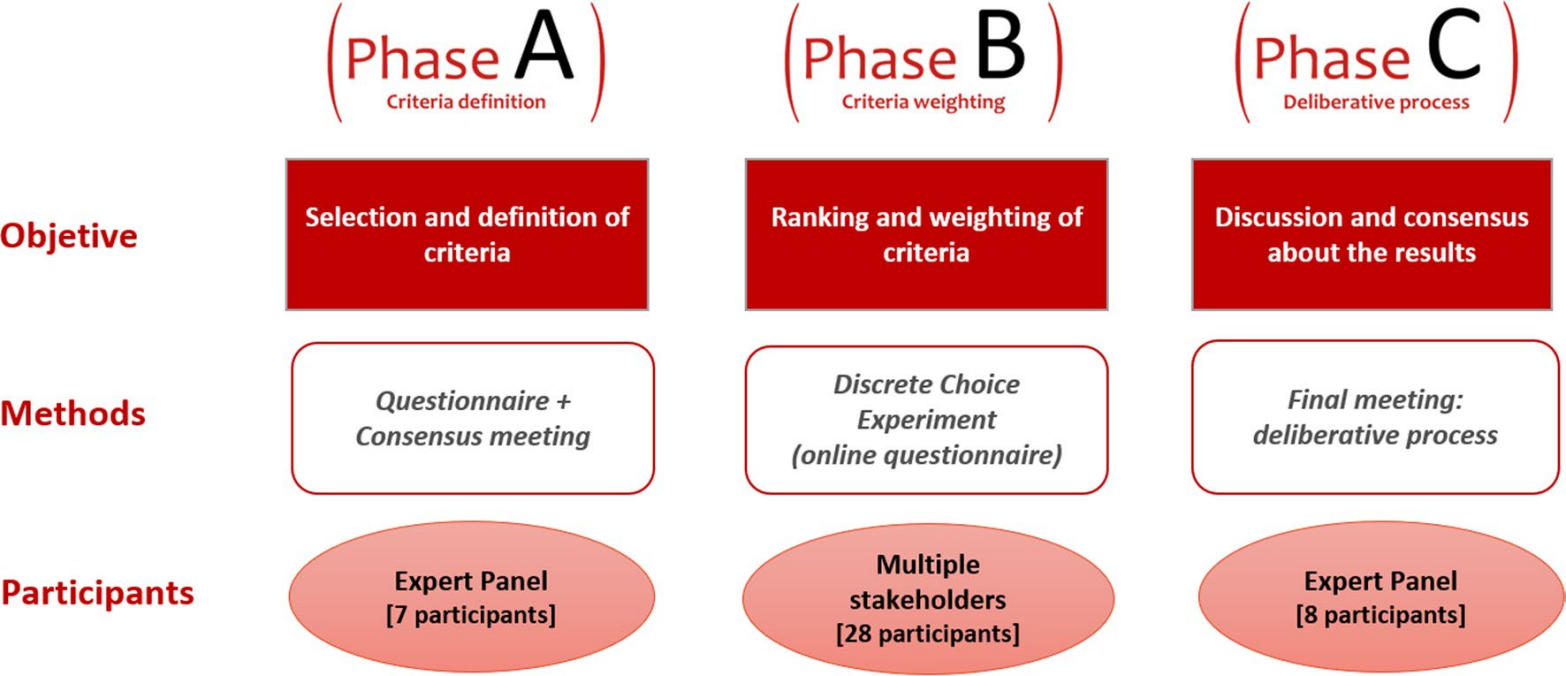
FinMHU-MCDA study: phases of the study

To identify possible reimbursement criteria for OMP, a literature review was conducted (July 2019) selecting different nationally and internationally published MCDAs. A search was done in the MEDLINE database through the PubMed search engine using specific terms of interest combined by Boolean operators (AND, OR), with no publication date limit (Fig. 2). We sought studies aimed at decision-making about orphan drugs and rare diseases that were conducted using MCDA methods and describing criteria for drug financing. The search identified a total of 80 publications. Of the articles identified, 22 were eliminated as duplicates or for being the same study conducted in different countries but with the same criteria. The other 58 publications were selected for full-text reading, after which we excluded 39 that did not fit the selection criteria, mainly due to not using MCDA methods or, if they did, for not being related to financing criteria. Finally, 19 references were included for the analysis and extraction of criteria (Fig. 1).

In the studies reviewed, the criteria corresponded to different factors related to orphan drugs, their indicated pathologies, and the patient population for which they are prescribed, including clinical and nonclinical variables. These criteria were characterized according to levels or categories. Each level defined or explained one of the characteristics or values that a criterion could present regarding an orphan drug, a rare disease, or a certain population. These levels could be quantitative or qualitative, they had to be exclusive and differentiated within that criterion in relation to the scope of the study; that is, the difference between whether a criterion was defined at one level or another could reflect a change in decision-making.

Based on the criteria identified in the literature, an online questionnaire was designed. It had an initial proposal of criteria and their levels, and was shared with the panel of experts for validation, with the option of modifying the criteria and suggesting additional ones, if considered. The answers to the questionnaires were shared during a face-to-face meeting of the panel of experts, where a consensus was reached regarding the list of criteria, including their designations, definitions, and levels (Table 1). This list of definitive criteria served as the basis for designing the next phase of the study.

**Table 1:** Reimbursement criteria for orphan drugs

Name of the criterion and definition	Levels	
*Population*		
1. Target population Number of patients affected by the disease who are candidates for treatment, according to prevalence and/or incidence	Prevalence < 0.2 per 10,000 inhabitants	
	Prevalence between 0.2 and 1 per 10,000 inhabitants	
	Prevalence > 1 but < 5 per 10,000 inhabitants	
2. Age of target population Age at the beginning of treatment of the disease	Nonpediatric	
	Pediatric	
*Disease*		
3. Disease severity Degree to which patient is affected	Mild	
	Moderate	
	Severe	
4. Economic burden of the disease Economic impact of the disease on the health system and society in general, considering the types of resources and costs involved ^a, b, c^	Low economic impact	
	Moderate economic impact	
	High economic impact	
*Treatment*		
5. Safety Adverse events due to treatment 5.1 Seriousness 5.2 Frequency	Serious AE	Frequent AE
	Nonserious AE	Infrequent AE
6. Availability of treatment alternatives Availability of different therapeutic options	No other therapeutic options	
	There are other options, but the current treatment improves health more than the alternatives	
	There are therapeutic options with similar characteristics	
7. Efficacy Expected clinical benefit or actual clinical benefit in the framework of a clinical trial	High benefit: curative or significant increase in survival	
	Moderate benefit: stabilization of the disease or improvement in quality of life	
	Low benefit: palliative or symptomatic	
8. Quality of evidence Credibility and robustness of evidence	Randomized controlled trial with comparator	
	Other types of clinical trials or with inappropriate comparator	
	Nonrandomized study	
9. Health-related quality of life Change in patient’s health-related quality of life due to the treatment received, associated with impaired mobility, personal care, daily activities, pain/discomfort, or anxiety/depression	Treatment improves health-related quality of life	
	Treatment does not modify health-related quality of life	
	Treatment decreases health-related quality of life	
*Economic evaluation*		
10. Cost of treatment Cost per patient per year^d^	< €100,000 per year	
	€100,000 to €300,000 per year	
	> €300.000 per year	
11. Costs avoided by treatment Reduction in costs derived from application of treatment, including medical costs^a^, non-medical costs ^b^, and indirect costs^c^	Avoids direct medical and nonmedical costs derived from the disease and indirect costs due to loss of productivity	
	Avoids direct medical costs derived from the disease	
	Does not avoid direct/indirect costs of the disease, or there is not enough information on avoided costs	
12. Cost-effectiveness Efficiency of a treatment, according to the criterion and the payers’ willingness to pay, evaluated by the incremental cost-effectiveness ratio expressed as cost per quality-adjusted life year gained from the intervention against a comparator or standard treatment	Cost-effective	
	Not cost-effective	

^a^Direct medical costs associated with the diagnosis, treatment, and management of patients with the disease

^b^Nonmedical direct costs derived from the disease (generally borne by the patient, caregiver, or social services)

^c^Indirect costs derived from the loss of productivity due to absenteeism/sick leave

^d^Cost per complete treatment in single-dose treatments

### Phase B: weighting and prioritization of the criteria

Once the potentially relevant criteria for decision-making about the financing of OMPs were agreed upon, an analysis was done using a preference elicitation technique. This aimed to identify which criteria were relevant for decision-making and to prioritize and weight them according to their relative importance.

The selected technique was a discrete choice experiment (DCE), which was developed based on the international recommendations of ISPOR [23, 24]. This method was performed using an online questionnaire addressed to the different stakeholders participating in the study, including the panel of experts from the first phase.

The questionnaire to compile the stated preferences of the participants was designed by combining criteria of different levels that were selected in phase A of the MCDA, by which we generated hypothetical financing situations or scenarios. Each scenario consisted of a unique combination of criteria and levels that described the characteristics of an orphan drug, a rare disease, and a hypothetical target population. Each question of the DCE offered two hypothetical financing scenarios, and the participants had to select the most favorable scenario for OMP reimbursement from the two hypothetical scenarios. To establish the statistical significance of criteria relevant to those decisions, the minimum number of pairs of scenarios that needed to be included in the questionnaire was calculated through an orthogonal design using the “Support.Ces” package.

Two multinomial logit models (general and reduced) were adjusted to analyze questionnaire responses and determine the relevance of the criteria and their levels according to the preferences of the participants. With the general model, we determined the strengths of preferences over the criteria from the coefficients obtained for each criterion. The reduced model determined which criteria were relevant to the selection of one financing scenario over another during the DCE. This reduction of criteria was performed using the Bayesian Akaike information criterion (AIC), eliminating the less informative criteria that were not decisive (p-values > 0.05). Considering *n* evaluated criteria, the relative importance (W_D_) was calculated using the following formula given the regression coefficients of each criterion:

$$V_{D} = \frac{{\left| {Coef_{D} } \right|}}{{SE_{D} }}\quad W_{D} = \frac{{V_{D} }}{{\mathop \sum \nolimits_{i = 1}^{n} V_{Di} }} \times 100$$

Coef: coefficient; SE: standard error.

All statistical analyses were performed using R software (version 3.2.3, R Foundation for Statistical Computing, Vienna, Austria) [25].

### Phase C: deliberative process

In this last phase of the MCDA, the results of the DCE on the prioritization and weighting of the criteria according to their relative importance were presented in the face-to-face meeting with the panel of experts, with the objective of discussing and interpreting the results through a deliberative process. The conclusions resulting from this process are reflected in the Discussion of this article.

## Results

### Selection and definition of levels

From the 19 publications selected in the literature review, 225 reimbursement criteria related to the treatment, the disease, and the affected population were identified. Of these, 125 were repeated exactly in more than one publication, resulting in a total of 100 different criteria. This list was simplified to 77 due to similarities between them. Finally, these criteria were grouped into 17 major concepts (Fig. 2). Based on these concepts, an initial proposal of 14 OMP reimbursement criteria for this study was designed, including its categorization on different levels, for review by the panel of experts. After completing the questionnaire, the initial meeting, and several specific reviews with the panel of experts, the 12 final reimbursement criteria of OMPs were established (Table 1). The criteria “Authorized indications” and “Budgetary impact” were removed from the final list, despite being criteria that frequently appear in this type of analysis. The panel of experts considered that the number of indications would not be a relevant criterion for decision-making since reimbursement is granted at indication level. Regarding the budgetary impact, the possibility of double counting was brought up, since this criterion includes both the population of patients to be treated and the cost of treatment, criteria already included individually in the analysis.

### Screening, prioritization, and weighting of criteria

Based on the 12 definitive reimbursement criteria, the DCE questionnaire was designed. Due to methodological limitations and to avoid an excessive number of questions that would hinder correct completion, the “Safety” criterion was divided in two: one considering the seriousness (serious/nonserious) and one considering the frequency of adverse events (frequent/infrequent). From the combination of the criteria and their levels, 36 was the minimum number of pairs of hypothetical scenarios necessary to establish the questions of the online questionnaire. This questionnaire was sent to a total of 89 relevant people in the field of rare diseases, of whom 29 (32.6%) completed the questionnaire. One questionnaire had to be excluded from the analysis because it was not complete.

The analysis of the DCE responses showed that 9 of the 13 criteria (counting the double safety criterion) were relevant to decision-making (Table 2). From the whole cohort of participants, economic burden of the disease, frequency of adverse events (safety), cost of treatment, and cost-effectiveness were the criteria that were the least informative at the time of selecting one financing scenario over another; that is, other criteria were more relevant in the choice.

**Table 2:** Results of the discrete choice experiment

General model	All participants (n = 28)						Patient Association (n = 4)	Physicians (n = 6)	Health economics (n = 7)	Hospital pharmacy (n = 5)	Health Authorities (n = 6)
	Beta (coefficient)	Standard error	Z-value	p. value	Odds ratio	Rel. Imp (%)	Rel. Imp (%)	Rel. Imp (%)	Rel. Imp (%)	Rel. Imp	Rel. Imp (%)
HRQoL	− 1.011	0.069	− 14.742	< 0.001	0.364	**23.53**	**14.27**	**20.55**	**25.11**	**22.35**	**21.83**
Efficacy	− 0.614	0.067	− 9.175	< 0.001	0.541	**14.64**	**13.23**	**15.05**	**8.86**	**17.70**	**10.73**
Availability of treatment alternatives	− 0.552	0.065	− 8.466	< 0.001	0.576	**13.51**	**11.00**	**9.92**	**6.00**	**16.43**	**19.39**
Disease severity	0.514	0.065	7.907	< 0.001	1.672	**12.62**	**13.93**	**11.62**	**14.82**	**8.89**	5.27
Avoided costs	− 0.452	0.064	− 7.020	< 0.001	0.636	**11.21**	**11.55**	**10.45**	**13.06**	**9.27**	**6.90**
Age of target population	− 0.499	0.103	− 4.854	< 0.001	0.607	**7.75**	**6.55**	**8.20**	2.15	**8.22**	**10.16**
Safety (seriousness of AE)	− 0.303	0.103	− 2.956	0.003	0.738	**4.72**	**8.70**	**5.49**	4.15	1.50	1.10
Quality of evidence	− 0.151	0.063	− 2.392	0.017	0.860	**3.82**	3.91	**7.21**	2.44	4.50	1.05
Target population	− 0.125	0.064	− 1.952	0.051*	0.883	**3.12**	2.62	0.38	2.13	3.61	**7.26**
Economic burden of the disease	0.103	0.065	1.568	0.117	1.108	2.50	3.15	3.78	2.97	2.43	2.78
Cost of treatment	0.072	0.066	1.086	0.278	1.074	1.73	2.34	2.57	**4.72**	0.79	**4.88**
Cost-effectiveness	− 0.054	0.103	− 0.518	0.604	0.948	0.83	7.57	2.83	**9.46**	2.04	**6.15**
Safety (AE frequency)	− 0.002	0.103	− 0.017	0.986	0.998	0.03	1.19	1.73	4.12	2.25	2.52

*p = 0.042 (< 0.05) in the reduced model. In bold, relevant criteria for decision-making. AE: adverse events; HRQoL: health-related quality of life; Rel. Imp.: relative importance

According to the results obtained, preference was given to a scenario that financed OMPs for diseases with a target population of lower prevalence, of nonpediatric age, and prioritizing the most severe pathologies. It was important that the orphan drug presented nonserious versus serious adverse events and that no other treatment options were available. In terms of the efficacy criteria, treatments that were curative or that significantly increased survival were preferred over other benefits, as were treatments that had high-quality evidence from controlled clinical trials with comparators. In addition, treatments that improved the health-related quality of life of patients and that avoided higher disease costs were prioritized, including both direct medical and nonmedical costs and indirect costs associated with loss of productivity.

Considering the reimbursement criteria all together, the relative importance of each criterion with respect to the rest was determined. The impact of treatment on health-related quality of life was the criterion with the greatest weight in decision-making, with 23.53% relative importance, followed by the benefit obtained with efficacy (14.64%), availability of treatment alternatives (13.51%), disease severity (12.62%), and avoided costs (11.21%). The rest of the criteria had a relative importance of less than 10%. For each group of stakeholders, the relative importance of each of the evaluated criteria was also obtained (Table 2). For each group, the criteria of health-related quality of life, efficacy, availability of treatment alternatives, and avoided costs were relevant. In the Health Authorities and Health Economics groups, the three criteria related to economic evaluation (cost of treatment, avoided costs, and cost-effectiveness) were relevant to decision-making, making up more than 25% of the decision in the group of Health Economists. The impact of treatment to health-related quality of life was over 20% in all groups, except for the Patient Associations group, for whom the five most important criteria presented a more uniform distribution.

## Discussion

The FinMHU-MCDA study has established, based on a multi-stakeholder and multi-disciplinary panel of experts in the field, the relative importance of different criteria for the reimbursement process for OMPs in Spain. These criteria and their relative importance weights can serve as a starting point to guide and inspire health authorities and the rest of decision makers involved to promote the development of a specific framework of multicriteria evaluation and move forward to a structured plan for the evaluation of OMPs. All of this with the aim of providing greater clarity and transparency in the access and reimbursement of OMPs in Spain. These criteria and relative importance weights could be further updated to reflect the current situation and the preference of the stakeholders involved in the process. Additionally, this initiative could be considered as a national reference and also contribute for a more uniform OMPs assessment at regional/local levels. In addition, this work establishes a reference framework for the development of future MCDA with direct application on OMPs in specific rare diseases.

The evaluation, selection, and financing of OMPs should be considered in a differentiated way that takes into account the particularities inherent to these treatments with respect to medicines with indications other than rare diseases. For the most part, evidence supporting the efficacy of these treatments is more difficult to gather at the time of marketing authorization than for other drugs because of the conditional and/or accelerated approval granted to benefit the patients receiving these treatments and the limited number of patients due to the low prevalence of these pathologies. Additionally, rare diseases can be serious pathologies that are life-threatening or chronically debilitating and have few or no treatment options. Many of the treatments cannot cure the disease but do improve the general condition of the patients in aspects that can translate into a better quality of life. The limited number of patients to whom they are directed would downplay the cost of treatment compared to other factors. The importance of the cost-effectiveness ratio of these treatments could also be disregarded, since many of the drugs financed are not usually cost-effective [7, 17, 18], so other treatment considerations determine the amount of public financing and their inclusion/exclusion in the therapeutic arsenal. These particularities, which are evident to a greater or lesser extent during the evaluation of the financing of drugs for rare diseases, were reflected in the participants’ preferences regarding the reimbursement criteria. Their responses reflect a high importance of how the treatment improves the quality of life of the patient, without neglecting the efficacy or disease severity or whether there are treatment options, while dismissing the economic criteria, whose relative importance could be greater when evaluating other non-orphan, more traditional, treatments. However, the costs avoided by the treatment would be more relevant because most rare diseases are associated with a significant economic burden, in both direct and indirect costs, the latter comprising a considerable proportion of the total costs [26].

One striking result of this study was the panel’s tendency to prefer a financing scenario for a nonpediatric population. One of the possible interpretations proposed by the expert panel was due to the naming of the criterion levels (pediatric/nonpediatric), which could be difficult to differentiate from a decision context with more criteria involved. Another could be the fact that among clinical decision-makers, there was only one pediatrician, and many rare disease specialists are internists who treat the adult population. In this sense, during the deliberative process, the modification of the levels of the criteria was suggested to distinguish them using different terms such as adults and children or adults and pediatrics (children, adolescents).

One of the strengths of the study is the multi-stakeholder perspective. Although health authorities make the decisions about reimbursement and access to the medications in the National Health System, it is of great importance to facilitate the participation of all sectors with decision-making capacity and opinion-leading positions on rare diseases so as to represent the different existing perspectives, including those of the management/payers, health professionals, and, especially in rare diseases, patients, who bring a unique perspective and are the best experts in their pathologies. Therefore, for the decision to be legitimate, a multi-stakeholder perspective must be incorporated in the development of tools that facilitate decision-making, with MCDA being one of the best approaches to facilitate this participation [27]. Another important element of this study, that strengthens these MCDA studies is the use of decompositional methods for eliciting preferences, such as the DCE, where the criteria are evaluated together [22]. Preferences over criteria are extracted indirectly through the choice between two hypothetical alternatives that embody a set of criteria, as would be done in reality. This allows us to evaluate how the preference for a criterion behaves in the presence of one or another criterion, showing its relative importance. At the same time, one of the possible limitations of this method arises when the number of criteria to consider increases beyond a certain point; too many criteria will increase the number and complexity of the DCE questions [22]. Another limitation of MCDA is that the results will always depend on the number of participants and the proportion of participants belonging to each group of stakeholders. In this sense, we aimed to reach as many participants, and with the most balanced distribution between groups, as we could, but this proportion was finally determined by the acceptance of the invited experts. Ethical criteria related to equity, fairness and justice, population specific interests or priorities, vulnerable populations appear in MCDA frameworks. However, these aspects are often used as contextual criteria, not being included in the core framework where quantitative weights are assessed. In this sense, a potential limitation of our study in the selection and definition of the MCDA criteria could be the non-inclusion of these ethical criteria explicitly, an aspect of great importance in the evaluation of rare diseases.

In the last decade, several analyses have been published evaluating and assessing the criteria that could be considered in the field of OMPs. A recent scoping review [17] compiled the most relevant experiences in recent years regarding the evaluation of orphan drugs in terms of coverage and drug reimbursement in several countries using MCDA, among other approaches [28–40]. In these studies, a group of 10 criteria was identified that best reflected the particularities of rare diseases as well as the preferences of the different stakeholders regarding the reimbursement decisions for OMPs. The participants in these projects were decision-makers, clinicians, and patient representatives. The criteria of disease severity, available treatment options, and comparative efficacy and safety were the criteria that appeared most frequently, maintaining a high relative importance in all these studies. In contrast, criteria such as innovation, a single indication for the drug, manufacturing complexity, and criteria related to health economics (cost-effectiveness, budgetary impact) appeared less frequently and were considered secondary criteria. The rarity of the disease was a relatively frequent criterion but of low relevance in all included studies. In the studies analyzed, aspects related to the efficacy and therapeutic benefit of the treatment, and the disease severity, were usually considered a priority over less important aspects related to the manufacturing process, technological innovation, or economics, aligning with what is reflected in the FinMHU-MCDA study. Unmet needs were also identified as an important criterion given the lack of knowledge about the natural history of these diseases, their diagnosis, and therefore the absence or scarcity of treatment options [17].

More recently, an MCDA has been performed at the international level that, like the present study, evaluated the relative preferences for the criteria to be included in a decision framework for OMPs, considering a multi-stakeholder perspective via a sample of 120 experts and an expert focus group [41]. That study included stakeholders from different fields, such as the pharmaceutical industry and academia, these two being the majority. It followed a direct rating method based on a total distribution of 100% relative importance among 13 criteria. In both groups of experts, the efficacy and safety of treatment and the severity and unmet needs of the disease were considered the most important criteria, in line with the FinMHU-MCDA study, especially when considering the results of the larger sample of stakeholders. One aspect to highlight in that study was that drug price was not included, because it was considered as a criterion with great influence when evaluating the preferences for the other criteria. However, this criterion was included in the FinMHU-MCDA as it was considered to be important in the decision. Also highlighted in that study was the usefulness of having a discussion group, as the deliberative process generated additional insights regarding the importance of criteria [41]. Regarding the methods used, some studies have used the DCE as a method of eliciting social preferences regarding the reimbursement of orphan drugs [42–45]. One of them, conducted in several European countries, including Spain, determined that the most preferred criteria were the cost of treatment, improvement in health, value for money, and availability of treatment alternatives [44].

In Spain, there have been some initiatives to evaluate drugs through MCDA methodology [46–48] an MCDA has recently been published to encourage the evaluation, positioning, and decision-making regarding OMPs at the national level [46]. In it, 15 criteria were selected for decision-making about OMPs based on the EVIDEM framework and methodology [47]. In this case, the weighting of the criteria was obtained from a previous, nonspecific study of OMPs conducted with 98 health professionals working in drug evaluation committees. The most important criteria were disease severity, comparative efficacy, quality of evidence, and comparative safety/tolerability. Other Spanish studies have used the MCDA method to validate and implement an OMP evaluation framework at the regional level, with evaluators and decision-makers from the Catalan Health Service (CatSalut) [36, 48]. In these studies, 10 quantitative and four contextual criteria were evaluated using the EVIDEM framework, the most important being disease severity, unmet needs, comparative effectiveness, and comparative safety/tolerability. The importance of the present study was that it was performed in all of its phases with a great variety of type of stakeholders related to OMPs bringing a comprehensive approach and conducting a discrete choice experiment, which improves methodological rigor in these studies.

Despite the different methods and the geographical scope, there are similarities between all the aforementioned studies and the FinMHU-MCDA study with respect to the selection of criteria, many of them appearing frequently. Regarding their relative importance, and although there is greater variability between studies, many of the most important criteria in the FinMHU-MCDA study also appear among the most relevant of the studies.

## Conclusions

To ensure adequate OMP access and reimbursement, it is necessary that decisions be arrived at through a process in which the preferences over the financing criteria are transparent and explicit, in which all types of agents involved in the field of rare diseases are incorporated, and in which practical tools that favor this process, such as MCDA, are applied.

From a multi-stakeholder perspective, the financing of an orphan drug will be conditioned by its effect on the health-related quality of life, the degree of its therapeutic benefit, and the availability of other treatment options. The severity of the rare disease for which the OMP is indicated is also relevant, as is the extent to which the treatment can avoid the costs associated with this pathology.

## Data Availability

The datasets generated or analysed during this study are available on request from Pharmacoeconomics & Outcomes Research Iberia (PORIB), under authorization of the data owner. The data are not publicly available due to restrictions related to confidentiality.

## Abbreviations

AEMPS: Spanish Medicines Agency *(Agencia Española de Medicamentos y Productos Sanitarios)*
AIC: Akaike Information Criterion
DCE: Discrete Choice Experiment
EMA: European Medicines Agency
FEDER: Spanish Alliance on Rare Diseases *(Federación Española de Enfermedades Raras)*, ISPOR: International Society for Pharmacoeconomics and Outcomes Research
OMPs: Orphan Medicinal Products
